# Complementary feeding at 4 versus 6 months of age for preterm infants born at less than 34 weeks of gestation: a randomised, open-label, multicentre trial

**DOI:** 10.1016/S2214-109X(17)30074-8

**Published:** 2017-04-07

**Authors:** Shuchita Gupta, Ramesh Agarwal, Kailash Chandra Aggarwal, Harish Chellani, Anil Duggal, Sugandha Arya, Sunita Bhatia, Mari Jeeva Sankar, Vishnubhatla Sreenivas, Vandana Jain, Arun Kumar Gupta, Ashok K Deorari, Vinod K Paul, Investigators of the CF trial, Chandra Kumar Natarajan, Ajay Singh, Reena Kuriakose, Faizan Mujeeb, Kanaklata Gupta, Farah Khan, Sukhram Babu, Garima Dhankar, Somi Suresh, Anne Therasa, Pawan Kumar Popli, Ramesh Sharma, Lalit Gupta, Brijesh Kumar, Vikas Yadav, Chander Prakash Yadav, Pratibha Gupta, Nisha Rani, Meenakshi, Sant Lal

**Affiliations:** a All India Institute of Medical Sciences, Delhi, India; b Vardhman Mahavir Medical College and associated Safdarjung hospital, New Delhi, India; c Kasturba Hospital, Delhi, India

## Abstract

**Background:**

Evidence on the optimal time to initiation of complementary feeding in preterm infants is scarce. We examined the effect of initiation of complementary feeding at 4 months versus 6 months of corrected age on weight for age at 12 months corrected age in preterm infants less than 34 weeks of gestation.

**Methods:**

In this open-label, randomised trial, we enrolled infants born at less than 34 weeks of gestation with no major malformation from three public health facilities in India. Eligible infants were tracked from birth and randomly assigned (1:1) at 4 months corrected age to receive complementary feeding at 4 months corrected age (4 month group), or continuation of milk feeding and initiation of complementary feeding at 6 months corrected age (6 month group), using computer generated randomisation schedule of variable block size, stratified by gestation (30 weeks or less, and 31–33 weeks). Iron supplementation was provided as standard. Participants and the implementation team could not be masked to group assignment, but outcome assessors were masked. Primary outcome was weight for age *Z*-score at 12 months corrected age (WAZ_12_) based on WHO Multicentre Growth Reference Study growth standards. Analyses were by intention to treat. The trial is registered with Clinical Trials Registry of India, number CTRI/2012/11/003149.

**Findings:**

Between March 20, 2013, and April 24, 2015, 403 infants were randomly assigned: 206 to receive complementary feeding from 4 months and 197 to receive complementary feeding from 6 months. 22 infants in the 4 month group (four deaths, two withdrawals, 16 lost to follow-up) and eight infants in the 6 month group (two deaths, six lost to follow-up) were excluded from analysis of primary outcome. There was no difference in WAZ_12_ between two groups: −1·6 (SD 1·2) in the 4 month group versus −1·6 (SD 1·3) in the 6 month group (mean difference 0·005, 95% CI −0·24 to 0·25; p=0·965). There were more hospital admissions in the 4 month group compared with the 6 month group: 2·5 episodes per 100 infant-months in the 4 month group versus 1·4 episodes per 100 infant-months in the 6 month group (incidence rate ratio 1·8, 95% CI 1·0–3·1, p=0·03). 34 (18%) of 188 infants in the 4 month group required hospital admission, compared with 18 (9%) of 192 infants in the 6 month group.

**Interpretation:**

Although there was no evidence of effect for the primary endpoint of WAZ_12_, the higher rate of hospital admission in the 4 month group suggests a recommendation to initiate complementary feeding at 6 months over 4 months of corrected age in infants less than 34 weeks of gestation.

**Funding:**

Indian Council of Medical Research supported the study until Nov 14, 2015. Subsequently, Shuchita Gupta's salary was supported for 2 months by an institute fellowship from All India Institute Of Medical Sciences, and a grant by Wellcome Trust thereafter.

## Introduction

Exclusive breastfeeding for 6 months followed by complementary feeding for term infants is a standard recommendation by WHO, widely endorsed and accepted by the global community. However, none of the organisations including WHO,^1^ the European Society of Paediatric Gastroenterology, Hepatology and Nutrition (ESPGHAN),^2^ or the American Academy of Pediatrics^3^ provide evidence-based guidelines with respect to the optimal time of initiation of complementary feeding in preterm infants who are at a much higher risk of postnatal growth restriction than full-term infants.^4^, ^5^ The only available guidelines are from the UK, and are based on a single, non-systematic review of primarily physiological studies.

Extrapolating the recommendation for full-term infants to initiate complementary feeding at 6 months of age to preterm infants is dependent on two major questions: what does 6 months refer to in a preterm infant—chronological (postnatal) age or corrected age? Health-care providers generally use corrected age for monitoring the physical growth and development of preterm infants. Secondarily, if we assume that 6 months refers to the corrected age, should these infants not start complementary feeding earlier than their full-term counterparts—for example, at 4 months corrected age instead of 6 months corrected age (appendix p 1)? Preterm infants have higher energy requirements compared with full-term infants,^6^, ^7^ and it is not known how long in infancy milk feeds alone (breastmilk or formula) are sufficient to meet their requirements. Most complementary foods provide higher calorie density compared with milk feeds, and can make up for the energy gap between increased requirements of preterm infants, and limited supply from milk feeds. Therefore, an earlier introduction of complementary feeding in preterm infants than is recommended for full-term infants might help improve their growth.

**Research in context**

**Evidence before this study**

We searched MEDLINE and Cochrane Central Register of Controlled Trials (CENTRAL) up to 6 Feb 2016, limited to human studies but with no restriction of language or publication date, using the terms “complementary food” OR “complementary foods” OR “complementary feeding” OR “supplementary feeding” OR “weaning” OR “weaning foods” OR “beikost” OR “semisolids” OR “semisolid foods” OR “semisolid food” OR “semisolid feeds” OR “infant feeding” OR “infant diet” OR “infant food” OR “infant foods”. We found 22723 articles on MEDLINE and 3667 on CENTRAL. After screening the title and abstracts and excluding duplicates, we reviewed the full text of 50 potentially relevant studies. We also searched the reference list of all these 50 studies to identify any other relevant studies.

Inclusion criteria for the Review were: any randomised, quasi-randomised trial or observational study investigating different time or age at initiation or introduction of complementary feeding in preterm infants. Complementary feeding was defined as initiation of semisolid, soft or solid foods other than breast, formula, or animal milk. We therefore excluded any studies enrolling full term infants or those where the term ‘weaning’ was used to indicate transition from breastfeeding to formula or animal milk rather than to semisolid, soft, or solid foods.

On full text review and reference list search, we identified three studies eligible for this Review—one randomised trial, one prospective observational study and one study that was a secondary analysis of data from original unrelated studies. The first study randomly assigned all preterm infants of less than 37 completed weeks gestation (n=68) to receive complementary feeding after 13 weeks of postnatal age if weighing at least 3·5 kg; or after 17 weeks of postnatal age if weighing at least 5·0 kg. The intervention, ie, initiation of complementary feeding in both groups, was also subject to parental perception of infant being ready to accept complementary feeding. The 13 week group also received a co-intervention in the form of energy and protein-dense food. The study did not find any significant difference in the SDs of length (0·2, 0·2 *vs* −0·1, 0·3), weight (–0·7, 0·2 *vs* −0·8, 0·2) and head circumference (–0·5, 0·2 *vs* −0·5, 0·2) between the groups. However, there was some improvement in length gain per week from birth to 12 months corrected age (mean cm per week, SD: 5·1, 0·07 *vs* 4·9, 0·10; p=0·04) and change in mean length SDs in the early weaning group (mean, SD: −1·1, 0·2 at birth to 0·2, 0·2 at 12 months corrected age) when compared with late weaning group (mean, SD: −1·0, 0·2 at birth to −0·1, 0·3 at 12 months corrected age).

The second study followed preterm infants from birth until 12 months corrected age, and recorded the time of initiation of complementary feeding based on information on introduction of 12 prelisted food items. Introduction of 4 or more items (n=203 infants) compared with that of less than 4 items (n=54 infants) by 17 weeks corrected age was associated with 3·5 times higher risk for developing eczema at 12 months corrected age.

The third study was a secondary analysis of data with respect to introduction of solid foods at ≤12 weeks or >12 weeks on growth parameters of preterm infants, from two previous trials—one on post-discharge formula *vs* full term formula (published), and another unpublished. The study did not find any difference with regard to introduction of solid foods ≤12 weeks or >12 weeks on weight gain in kg (mean, SE: 8·25, 0·05; n=365 *vs* 8·27, 0·07; n=102), length gain in cm (70·3, 0·16; n=362 *vs* 70·6, 0·2; n=102) and head circumference gain in cm (45·8, 0·08; n=364 *vs* 45·8, 0·12; n=102) between 12 weeks and nine months corrected age, or between 12 weeks and 18 months corrected age (data not reported). There was no difference in the prevalence of atopy, lower respiratory tract infection, gastroenteritis, sleep duration, or waking at night between the two groups.

**Added value of this study**

Our study provides level-1 evidence with regard to the timing (4 *vs* 6 months of corrected age) for initiation of complementary feeding in preterm infants less than 34 weeks of gestation, a group most susceptible to postnatal growth restriction. It shows that early initiation of complementary feeding at 4 month compared with 6 months of corrected age does not improve growth of preterm infants at corrected age of 12 months. It also does not result in a difference in neurodevelopment outcomes, body composition, bone mineralisation, and any marker for metabolic syndrome like insulin resistance, lipid profile, and blood pressure in infancy. Rather, earlier initiation of complementary feeding at 4 months corrected age increases the risk of hospital admission due to concurrent morbidities, predominantly diarrhoea and lower respiratory tract infections. In both groups, dietary patterns remain poor and body iron stores remain depleted despite iron supplementation until 12 months of corrected age.

**Interpretation**

Our study suggests that 6 months of corrected age should be preferred over 4 months of corrected age for initiation of complementary feeding in preterm infants less than 34 weeks of gestation. Findings of clinically significant iron deficiency despite iron supplementation and poor dietary patterns in infancy will guide further research.

We did a systematic review to answer this crucial question but found only one low quality randomised trial addressing the issue.^8^ We therefore chose to test the hypothesis that initiation of complementary feeding (defined as semisolid, soft, or solid foods other than breastmilk, formula, or animal milk) at 4 months compared with 6 months corrected age in infants less than 34 weeks of gestation will increase their weight for age *Z*-score at 12 months corrected age (WAZ_12_) by 0·5 standard deviation score (SDS; around 500 g, based on WHO-Multicentre Growth Reference Study [MGRS] growth standards).^9^ We also investigated neurodevelopment, body composition, bone density, and early markers of metabolic syndrome as there is little evidence on the links between infant feeding and later health, and sought to characterise potential effects of the nutritional intervention on some markers of the same.

## Methods

### Study design

We did this randomised, open-label, parallel group trial at three public health facilities; the All India Institute of Medical Sciences (AIIMS), Vardhman Mahavir Medical College associated Safdarjung hospital, and Kasturba hospital in New Delhi, India. All three sites provide tertiary care neonatal services to inborn neonates. Neonatal intensive care is provided to all infants less than 34 weeks of gestation, and exclusive breastmilk feeding is actively promoted (appendix p 2). Ethics approval was obtained from institutional ethics committees at all sites. The trial is registered with Clinical Trials Registry of India, number CTRI/2012/11/003149.

### Participants

Eligible patients were infants less than 34 weeks of gestation with no major malformation, residing within 60 km of study hospitals and not expected to move away from the study region within 1 year of birth. Infants requiring hospital admission from birth to later than 40 weeks postmenstrual age were excluded. Research team identified potentially eligible infants at birth, kept them in follow-up after discharge and called their caregiver by telephone at 4 months corrected age for enrolment into the study. Infants were excluded at this stage if they had already been started on complementary feeding. Initiation of complementary feeding was defined as intentional initiation of complementary food by family irrespective of amount, unintentional initiation but food given for more than 3 days, or that the total amount given provided more than 1% of recommended dietary allowance of calories for the infant. Written informed consent was obtained from the parents or legally acceptable representative.

### Randomisation and masking

Participants were randomly assigned (1:1 allocation) to the 4 month group, wherein parents or family were advised to initiate complementary feeding at 4 months corrected age; or to the 6 month group, in which they were advised to continue milk feeds until 6 months corrected age, followed by initiation of complementary feeding at 6 months corrected age. An independent person (MJS) provided computer generated random sequences, stratified for site and gestation (30 weeks or less and 31–33 weeks) with variable block size unknown to the research team involved in implementation. Allocation was concealed using sealed, opaque, sequentially numbered envelopes. The families and research team involved in implementation could not be masked to the allocation groups following randomisation, however, those assessing primary and secondary outcomes were masked. Twins and triplets were assigned to the same group.

### Procedures

Detailed descriptions of the study procedures can be found in the appendix (p 3). At 4 months corrected age, we randomly assigned the infants and did baseline anthropometric measurements and Dual Energy X-Ray Absorptiometry (DXA).

A single person counselled the families for initiation of complementary feeding at corrected age 4 months or 6 months, using uniform, prerecorded audio-visual counselling instructions in local language. The instructions were based on the WHO guidelines on complementary feeding of the breastfed child,^1^ and specified the desired frequency, amount, consistency, and texture of food, and principles of responsive feeding, hygiene, feeding during and after illness, and maintenance of breastfeeding. In the audio-visual presentation, we also included a demonstration on cooking common recipes of the region, which were prepared and standardised in terms of energy and other nutrient densities in accordance with the WHO guidelines. Additionally, families' queries were resolved through one-to-one counselling. The mothers were provided a handout of the instructions and suggested recipes in local language, and a set of uniform household utensils with known capacity to measure the ingredients and to feed the child.

We asked mothers to maintain a daily dietary record incorporating information on type, frequency and amount of food consumed by the child, and problems faced, if any. Mothers maintained this record for 4 weeks beginning from date of counselling for initiation of complementary feeding. We reinforced at each visit the supplementation of the infants with vitamin D (400 IU per day) and elemental iron (2–3 mg/kg per day), which was started from 2 weeks of age as part of the study hospitals' clinical policy. Subsequently, we called the infants for hospital visit at corrected age 5, 6, 7, 9, and 12 months to measure anthropometry, morbidity inquiry, and 24 hour dietary recall (appendix p 4). Additionally, we provided families in both groups with 24-h, 7-day per week need-based telephone support. At the 12 month visit, we did neurodevelopmental assessments, DXA, and fasting blood sampling as part of final outcome assessment. If an infant could not be brought to the hospital, the study team made a home visit to measure outcomes, except blood sampling and DXA. We measured compliance to intervention through telephone calls made on days 2, 7, 14, 21, and 28 at 4 months and again at 6 months of corrected age in both groups, which was also corroborated by the daily dietary record maintained by the mothers.

We defined infants as receiving allocated intervention if they were offered complementary feeding within 4 weeks of counselling for initiation of food, irrespective of amount consumed. An additional criterion in the 6 month group was that if complementary feeding was started before the scheduled 6 months corrected age, it should not have been started more than a week before 6 months corrected age. The intrauterine growth category at birth and the *Z* scores for weight, length, and head circumference at birth, discharge, and 40 weeks of postmenstrual age were based on revised Fenton's charts 2013,^10^ calculated using the provided anthropometric software for research data. Nutrient intakes were calculated from 24 dietary recalls using the nutritive value of Indian foods.^11^ We also calculated important dietary indicators related to infant feeding at 9 and 12 months of age based on the information recorded during the 24-hour dietary recalls based on WHO definitions (appendix p 4).^12^ We also noted the consistency and texture of food, which was based on expert assessment due to absence of standard criteria. *Z* scores for weight, length, head circumference, weight for length, and body–mass index at subsequent timepoints, (ie, 4, 5, 6, 7, 8, 9, and 12 months corrected age) were calculated using WHO-MGRS growth standards.^9^

### Outcomes

The primary outcome was WAZ_12_, based on WHO-MGRS growth standards.^9^ The infant was weighed using an electronic weighing scale (Seca, Germany; accuracy 5 g) and the weight was converted into respective *Z* score using WHO Anthropometric software (version 3.2.2, 2011), using corrected age. Secondary outcomes were any morbidity requiring hospital admission from the time of enrolment until 12 months corrected age, neurodevelopment, body composition, bone mineral content (BMC) and bone mineral density (BMD), insulin resistance in terms of HOMA-IR (HOmeostatic Model Assessment for Insulin Resistance), lipid profile, blood pressure, and serum ferritin at 12 months corrected age. Hospital admission was defined as admission to an inpatient facility for duration of 6 h or more or as inpatient death irrespective of duration of admission. A paediatrician verified the diagnosis for each episode by reviewing the case records and interaction with the treating physician where required. Repeat hospital admissions for an infant should have been separated by more than a week to count as separate episodes. Neurodevelopment was assessed by a single certified clinical psychologist using Developmental Assessment Scale for Indian Infants (DASII), a validated Indian adaptation of Bayley-II.^13^ Whole body composition, BMC, and BMD were assessed using DXA (Hologic DISCOVERY W, S/N 84879, version 13.1.1:7; software Apex Version 3.0). HOMA-IR was calculated using the formula:^14^

$$\text{HOMA-IR}=\frac{\text{fasting}\;\text{insulin}(\mu \text{U}/\text{mL})\times \text{fasting}\;\text{glucose}(\text{mmol}/\text{L})}{22.5}$$

Fasting (minimum 4 h) plasma glucose was measured by Enzymatic Colorimetric Test Method (GOD-PAP method) without deproteinisation based on enzyme glucose oxidase, using Glucose PAP Fluid Mono reagent (Centronic GmBH, Germany), on ROCHE Modular P-800 fully automatic analyser. Serum insulin and ferritin were measured by electrochemiluminescence immunoassay (ECLIA) using RocheCobase411 (Roche Diagnostics, Germany). A value of serum ferritin less than 12 was taken as cutoff for depleted iron stores.^15^ For lipid profile, fasting total cholesterol and triglycerides were assessed using enzymatic methods and high density lipoprotein by direct method, on ROCHE P-800 fully automatic analyser. Very low density lipoprotein was calculated using triglyceride value and low density lipoprotein was calculated using total cholesterol, high density lipoprotein, and triglyceride values using Friedewald equation.^16^ Blood pressure was measured using arm-type fully automatic digital blood pressure monitor (AG-SafeCHEK™ model AG1010; accuracy 3 mm Hg), based on the oscillometric method, using reusable blood pressure cuff (WelchAllyn® Flexiport™ infant size number 7). Additionally, serum C-reactive protein was measured using high sensitivity C- reactive protein Enzyme Immunoassay (BioCheck, USA). This was done to better reflect on the value of serum ferritin, which is also an acute phase reactant.

### Statistical analysis

We based our sample size estimates on the data on WAZ_12_ for infants less than 34 weeks of gestation from a birth cohort study of preterm infants from our own unit, which measured growth outcomes using WHO-MGRS growth standards,^16^ prospectively at 3 monthly intervals from birth till 18 months of age (WAZ_12_: 1·7 ± 1·5, n=15; personal communication, Sharma P).^17^ Assuming an effect size of 0·5 SDS, β of 0·9, and a two-sided α of 0·05, the sample size was 190 in each group. Accounting for 5% loss to follow-up, total sample size was calculated to be 400. The calculated sample size was further corroborated through a pilot study (infants in pilot phase were not included in this study).

Data were entered in duplicate in an online database developed in Visual Basic as front-end and MS SQL server as back-end with inbuilt range and logical checks, with audit trail. Analysis was done using STATA 11·0 (College Station, TX, USA), by intention to treat. Continuous variables were compared using Student *t* test for normally distributed and Wilcoxon rank-sum for non-normally distributed data. Proportions were compared using chi-square test. We constructed a Kaplan-Meir survival curve to depict the first episode of hospital admission in each group during the study period including available information on all infants, and calculated the hazard ratio between the two groups using Cox proportional hazard regression. We also used the Anderson-Gill model, which is counting process extension for Cox proportional hazard regression to calculate the hazard ratio between the two groups to account for multiple episodes of hospital admission for any infant.

A prespecified subgroup analysis was done by site, intrauterine fetal growth category (small-for-gestational-age, appropriate-for-gestational-age), gestational age at birth (<28 weeks, 28–30 weeks and 31–33 weeks), and type of feeding at randomisation (breastfed, non-breastfed, mixed fed). Generalised estimating equation analysis was used to compare anthropometry trend between the two random groups over time. We accounted for the clustering effect on the primary outcome due to corandomisation of twins and triplets by reanalysing the primary outcome after dropping multiple births (retaining first twin only) in both groups. Study was supervised by an independent Doctoral Committee that reviewed the processes every 6 months. An independent safety adviser also reviewed the data on mortality and morbidity.

### Role of funding source

The Indian Council of Medical Research had no role in study design, data collection, analysis, interpretation, or writing of the report. The corresponding author had full access to all the data in the study and had final responsibility for the decision to submit for publication.

## Results

**Figure 1:**
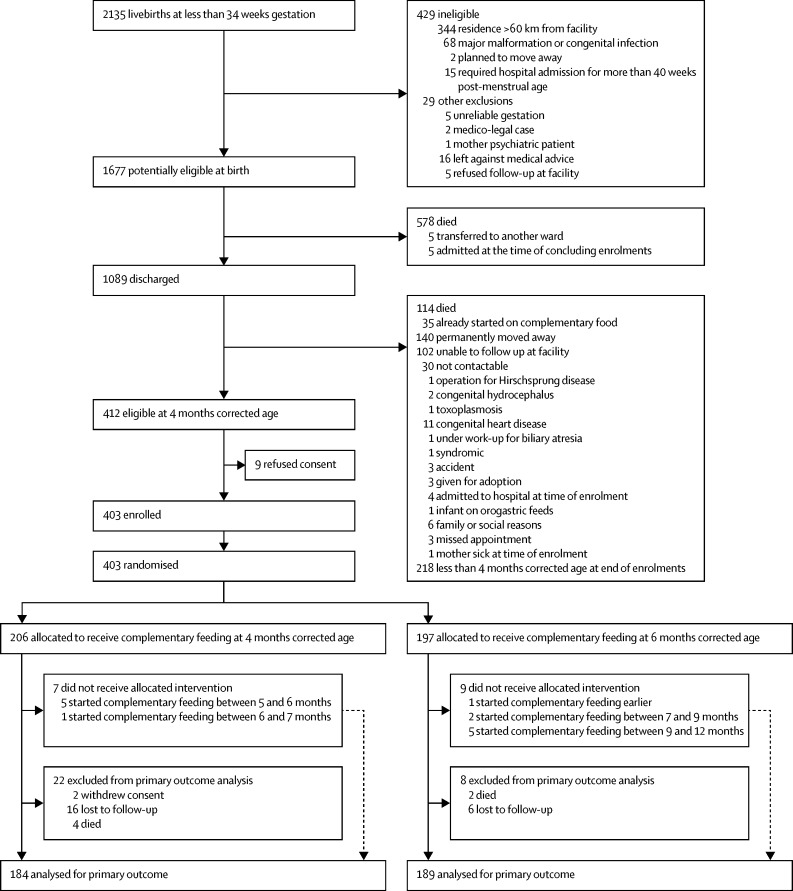
Trial profile

Between March 20, 2013, and April 24, 2015, we identified 2135 livebirths at less than 34 weeks gestation. 412 were eligible at 4 months corrected age, nine of whom refused consent, and 403 were randomly assigned—206 infants to the 4 month group and 197 infants to the 6 month group (figure 1). Overall, a total of 22 infants in the 4 month group (a pair of twins had consent withdrawn, 16 infants were lost to follow-up, and four died) and eight infants in the 6 month group (six infants were lost to follow-up and two died) were excluded from the analysis of the primary outcome. However, deaths were included in the analysis as secondary outcome.

196 of 203 infants in the 4 month group (two withdrew consent, one died before complementary feeding could be initiated) and 184 of 193 infants in the 6 month group (two died and two lost to follow-up before complementary feeding could be initiated) received the allocated intervention.

The baseline characteristics of study infants at birth and at randomisation were similar (table 1). Mean age at randomisation was 3·9 months corrected age in both the groups, and mean weight and WAZ were 5117 g in the 4 month group versus 5187 g in the 6 month group and −2·3 in the 4 month group versus −2·2 in the 6 month group. The proportion of infants receiving any breastfeeding was similar between two groups at all timepoints (appendix p 5). A high proportion of infants were receiving complementary feeding (irrespective of amount) 1 month after counselling for initiation of food (95·4% *vs* 90·7%; appendix p 5).

**Figure 2:**
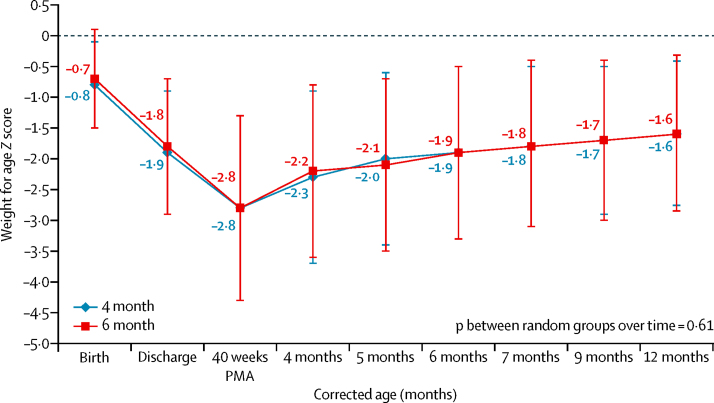
Change in weight for age *Z* score among study infants over time by study group. *Includes data only for infants who completed 12 month follow-up; data are mean (SD). PMA=postmenstrual age.

The primary outcome, WAZ_12_, was similar between the two groups (mean −1·6, SD 1·2 in the 4 month group *vs* −1·6, 1·3 in the 6 month group; mean difference 0·005, 95% CI −0·24 to 0·25; p=0·96) at mean corrected age of 12·2 months (table 2). The change in WAZ in study infants from birth until 12 months corrected age (p=0·61; figure 2) and specifically between 4 and 12 months of corrected age was similar (p value on generalised estimating equation [GEE] analysis, p=0·836). Mean weight (7794 *vs* 7846 g, p=0·65) at 12 months corrected age was also similar (appendix p 5). Additional growth data is provided in the appendix (pp 6–7).

**Figure 3:**
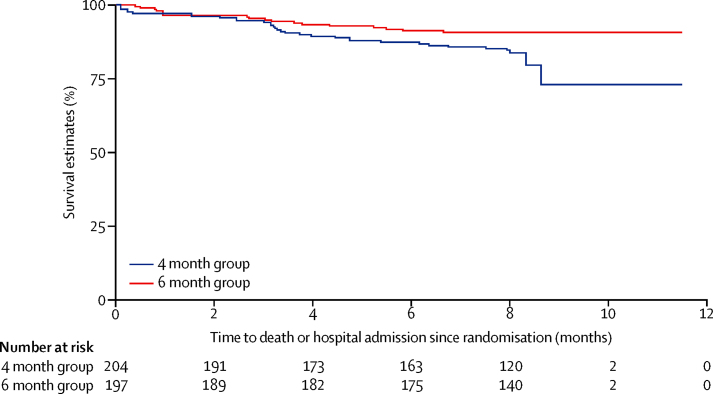
Corrected age at first hospital admission among study infants during the study period by study group

Six infants died during the study period; four of 203 in the 4 month group versus two of 197 in the 6 month group (risk ratio [RR] 1·9, 95% CI 0·4–10·5; table 2). 34 (18%) of 188 infants in the 4 month group required hospital admission during the study period, compared with 18 (9%) of 192 infants in the 6 month group (RR 1·9, 95% CI 1·1–3·3; p=0·01), with 2·5 episodes per 100 infant-months in the 4 month group versus 1·4 episodes per 100 infant-months in the 6 month group (incidence rate ratio 1·7, 95% CI 1·0–3·0; p=0·04, table 2). The Kaplan-Meier curve for the time to first episode of hospital admission showed that the mean first hospital admission for the 4 month group was earlier compared with the 6 month group (p=0·02), with the risk of hospital admission 48% lower in the 6 month compared with the 4 month group (hazard ratio [HR] 0·52, 95% CI 0·29–0·92; p=0·025) during the study period after adjusting for randomisation group, site, and gestation. The results remained similar when multiple episodes of hospital admission for any infant were taken into account using the Anderson-Gill model (0·56, 0·33–0·94; p=0·029, figure 3).

The motor and mental development quotients in the two groups were similar (table 2). There was no significant difference in body composition, BMC, BMD, lipid profile, HOMA-IR, blood pressure, or serum ferritin between the two groups. Median serum ferritin was 5·4 μg/dL (IQR 3·2–12·4) in the 4 month group and 5·7 (2·5–13·3) in the 6 month group (p=0·73), with almost two-thirds of infants in both groups obtaining a result less than 12 μg/dL (table 2).

A prespecified subgroup analysis by site, intrauterine fetal growth category, gestational age at birth, and type of feeding at randomisation (breastfed, non-breastfed, mixed fed) did not present any difference in the primary outcome (table 3).

Dietary diversity was acceptable in less than two-thirds of infants in either group (60·1% *vs* 55·0%, p=0·32; table 4). Acceptable minimum meal frequency was present in most infants (93·4% *vs* 92·6%; p=0·75). However, less than two-thirds of infants were receiving minimum acceptable diet. A high proportion of infants in both groups were bottle-fed (table 4).

## Discussion

Our study shows that initiating complementary feeding at an earlier age of 4 months compared with 6 months of corrected age resulted in similar WAZ scores and other growth outcomes, body composition, bone mineralisation status, iron stores (with iron supplementation), and markers of metabolic syndrome at 12 months corrected age. However, there was a higher risk of hospital admission in the group with earlier initiation, suggesting that initiation of complementary feeding at 6 months corrected age might be preferable to 4 months corrected age in infants born at less than 34 weeks of gestation.

This evidence can be considered robust, as the study was adequately powered to detect the outcome of interest and met all criteria for high internal validity: it was a randomised trial with groups being similar at the start, groups were treated similarly except for allocated intervention, no co-intervention was administered, the overall follow-up rate was high, analyses were by intention to treat and the outcomes were either blinded or objective.

To our knowledge, only one trial published earlier compared the effect of initiation of complementary feeding on growth of preterm infants. The study had several limitations and concluded that there was no difference in the anthropometric parameters between the two intervention groups at 12 months of age. Two other studies—one in full-term, healthy and one in low birthweight, exclusively breastfed infants—have compared the effect of initiation of complementary feeding at 4 versus 6 months of age.^18^, ^19^ Both studies showed that the intervention resulted in no significant difference in weight or length at 12 months of age, similar to our study. Unfortunately, we could not measure breastmilk intake in our study. However, we did note that in breastfed infants, energy intake per kg bodyweight (excluding breastmilk) was higher in the 4 month group compared with the 6 month group at all timepoints, although it was similar among non-breastfed infants (except for a small difference at 6 months corrected age; appendix p 9). Since there was no difference in growth parameters between the two groups at any timepoint, it is likely that the breastfed infants in the 6 month group increased their breastmilk intake and were deriving the additional energy from breastmilk. Studies using robust isotopic methods have also shown increased breastmilk intake in full-term exclusively breastfed infants who continue to remain breastfed until 6 months of age, and decreased intake among those who start on complementary feeding at 4 months of age.^20^, ^21^

The overall incidence of hospital admission in the study population was low, but infants in the 4 month group had more episodes of diarrhoea and lower respiratory tract infections until 12 months corrected age. Although these might be explained by potential contamination of complementary foods due to inadequate hygiene,^22^ a more biological rationale should also be considered. Breastmilk is known to confer immunological benefits to infants that are especially important for preterm infants,^23^ and breastmilk intake is likely to have been lower among infants in the 4 month group, as discussed earlier. Besides, the role of dietary exposures in shaping both short and long term immune function in infants might also be a factor.^24^, ^25^ Little difference in growth patterns between the two groups with little catch up growth in either group might explain similar body composition and similar markers of metabolic syndrome between the groups. The mean fat mass and % fat at 12 months of corrected age in either group were close to that reported in literature, suggesting that the biology of growth among this group of infants is similar across settings.^26^ However, we need to be cautious with whole body DEXA as a measure as it is not able to accurately establish the aberrant adiposity that might occur.

Poor dietary practices among all study infants despite counselling are more difficult to understand, especially as available evidence suggests that counselling helps to improve feeding practices.^27^ We postulate that the practices would possibly have been worse without counselling as the indicators obtained in our study were better than reported in literature from similar settings.^28^ It appears that to achieve the recommended dietary standards in this population requires a degree of behaviour change at the family level that is difficult to achieve, and requires innovative approaches to supplement the counselling. It is also important to consider that infant factors such as taste, preference, ability, or interest in taking feeds and pattern of eating could also have contributed to the dietary patterns seen in the study.

Another important concern revealed by the study is the greatly depleted iron stores, despite most infants having received iron supplementation. This is a puzzling finding, and since we had strictly ensured the compliance, it requires some explanation. Studies have shown that iron bioavailability from habitual Indian diets is low, due to high phytate and low ascorbic acid to iron ratios. Additionally, food matrix effect and food synergies specific to the local context could have been an issue.^29^, ^30^ We also presume that coadministration of iron supplements with food or milk could have resulted in decreased absorption of iron. Delayed cord clamping was not being practiced at any of the sites during the study period, and that could also be a reason in part. However, despite all of these postulations, this is a crucial outcome with a bearing on neurodevelopment of these vulnerable infants. Therefore, it is important to do further research to ascertain the adequacy of recommended doses of supplementation in preterm infants, factors that retard iron absorption, and possibly better iron preparations with enhanced bioavailability.

This study was done in a lower middle-income country setting, but we propose that the results could hold relevance even for high-income country settings for two main reasons. First, we observe that growth pattern of this group of infants is similar across settings, with universal postnatal growth restriction.^31^, ^32^ In this study, the parameters of fat mass at 12 months of age were close to that reported in the literature.^28^ Second, the feeding or dietary patterns in infancy are also similar with low rates of exclusive breastfeeding and inadequate complementary feeding practices.^33^, ^34^, ^35^ However, other factors that might limit the generalisation of results to other settings and would need prior consideration include differences between developing and developed country settings with respect to the birthweight of preterm infants, prevalence rates of intrauterine growth restriction in the population, practices with respect to use of post-discharge, nutrient-enriched formula or animal milk, breastfeeding rates, fortification of complementary foods, background infection rates, and sociodemographic factors such as maternal education and socioeconomic status, which would influence hygiene and compliance to dietary advice.

We also report that the mean postnatal age of study infants at intervention was 5·7 months (SD 0·3) in the 4 month group, and 7·9 months (SD 0·4) in the 6 month group. Since the 4 month group did not result in improved outcomes but increased hospital admissions, it follows that 6 months of postnatal age might not be preferred over 6 months of corrected age for initiation of complementary feeding in preterm infants.

The limitations of the study are the open-label design and differential loss to follow-up in the two allocation groups. However, the outcome assessors were masked to the allocation groups, and the baseline characteristics of infants lost to follow-up in either group were similar. The diagnostic utility of any neurodevelopmental assessment at 1 year of age is also inadequate, which might also be considered a limitation. There is a need for longer term follow-up of this cohort, and reassessment of their growth, development, micronutrient status, and markers of chronic disease.

Future research should focus on identifying an appropriate window, if one exists, to improve the postnatal growth of preterm infants. There is also an urgent need to identify the reasons for poor dietary patterns and greatly depleted iron stores despite supplementation in the preterm infants, and test potential interventions and institute preventive measures to target the same. Long-term follow-up of such infants, ideally till adulthood, is highly desirable.

## Figures and Tables

**Table 1:** Baseline characteristics

			**4 month group (n=204)**	**6 month group (n=197)**
Maternal age, years (SD)			26·9 (4·9)	26·4 (4·1)
Paternal age, years (SD)			30·5 (5·5)	30·1 (4·7)
Maternal education				
	Professional, graduate, post-graduate		58 (28·5%)	44 (22·3%)
	Intermediate, post-secondary diploma, high school		68 (33·3%)	72 (36·6%)
	Middle, primary		61 (29·9%)	55 (27·9%)
	Illiterate		17 (8·3%)	26 (13·2%)
	Family income, thousand Indian rupees per month (IQR)		10 (7–15)	10 (8–15)
Infant characteristics at birth				
	Gestation, weeks (SD)		31·7 (1·4)	31·5 (1·7)
	Gestation category			
		<28 weeks	4 (2·0%)	6 (3·1%)
		28–31 weeks	66 (32·4%)	68 (34·5%)
		32–33 weeks	134 (65·7%)	123 (62·4%)
	Birthweight, g (SD)		1479 (308)	1492 (344)
	Birthweight *Z* score (based on weight for gestational age at birth)*		–0·84 (0·71)	–0·73 (0·76)
	Birthweight category			
		<1000 g	13 (6·4%)	13 (6·6%)
		1000–1499 g	79 (38·7%)	77 (39·1%)
		≥1500 g	112 (54·9%)	107 (54·3%)
	Small for gestational age*		58 (28·4%)	54 (27·4%)
	Multiple births		55 (27·0%)	48 (24·3%)
	Female sex		109 (53·4%)	93 (47·2%)
	Antenatal steroids received		169 (85·4%)	169 (86·2%)
	Duration of NICU stay, days (IQR)		7 (4–12)	6 (4–12)
Infant characteristics at randomisation				
	Corrected age (months)		3·9 (0·1)	3·9 (0·1)
	Weight (g)		5117 (906)	5187 (928)
	Weight for age (*Z* score)		–2·3 (1·4)	–2·2 (1·4)
	Length (cm)		58·5 (3·1)	58·6 (3·2)
	Length for age (*Z* score)		–2·0 (1·4)	–2·0 (1·5)
	Head circumference (cm)		38·9 (1·6)	39·0 (1·4)
	Head circumference for age (*Z* score)		–1·7 (1·2)	–1·7 (1·2)
	Weight for length *Z* score		–0·96 (1·1)	–0·9 (1·1)
	BMI (kg/m^2^)		8·7 (1·2)	8·8 (1·2)
	BMI for age *Z* score		–1·5 (1·2)	–1·5 (1·2)
Body composition				
		Fat mass (g)	1259 (746), n=166	1242 (662), n=149
		Lean+BMC mass (g)	4346 (836), n=166	4462 (715), n=149
		Total mass (g)	5624 (965), n=166	5690 (985), n=149
		Percent fat (%)	21·7 (11·2), n=166	21·1 (9·3), n=149
	BMC (g)		95·7 (24·6), n=173	93·8 (23·0), n=156
	Bone mineral density (g/cm^2^)		0·170 (0·028), n=173	0·167(0·022), n=156
	Method of feeding			
		Only breastfeeding	104 (51·2%)	98 (49·8%)
		Mixed feeding (breastmilk and animal or formula milk)	61 (30·1%)	55 (27·9%)
		Exclusively top-fed (no breastfeeding, only animal or formula milk)	38 (18·7%)	44 (22·3%)

Data are n/N(%), or mean (SD), unless otherwise specified. NICU=neonatal intensive care unit. BMI=body–mass index. BMC=bone mineral content.

* Using Fenton growth charts 2013.

**Table 2:** Primary and secondary outcomes

			**4 month group**	**6 month group**	**Mean difference or risk ratio (95% CI)**	**p value**
Primary outcome						
	Weight for age (*Z* score)		–1·6 (1·2), n=184	–1·6 (1·3) n=189	0·005 (–0·24 to 0·25)	0·965
Secondary outcomes						
	Death		4/203*	2/197	1·9 (0·4 to 10·5)	0·685
	Hospital admission					
		Infants, n (%)†	34/188 (18·1%)	18/192 (9·4%)	1·9 (1·1 to 3·3)	0·014
		Episodes per infant-month (IR)	39/1590 (0·025)	23/1606 (0·014)	1·7 (1·0 to 3·0)‡	0·039
	Diagnosis					
		Diarrhoea (with or without dehydration)	11	6	..	..
		LRTI	16	11	..	..
		Both diarrhoea and LRTI	1	1	..	..
		Sepsis	3	1	..	..
		Other	7	3	..	..
		Unclear	1	1	..	..
Neurodevelopment						
	MoDQ_50_		84·0 (15·4), n=182	83·8 (14·0), n=184	0·2 (–2·9 to 3·2)	0·918
	MoDQ_50_<70		27 (14·8%), n=182	22 (12·0%), n=184	1·2 (0.7 to 2.1)	0·552
	MoDQ_97_		104·3 (18·6), n=182	102·6 (16·8), n=184	1·7 (–1·9 to 5·4)	0·351
	MeDQ_50_		89·0 (12·4), n=181	89·2 (11·5), n=184	–0·3 (–2·8 to 2·1)	0·786
	MeDQ_50_<70		12 (6·6%), n=181	12 (6·6%), n=182	1·0 (0.5 to 2.2)	0·713
	MeDQ_97_		108·7 (18·4), n=181	109·0 (14·9), n=184	–0·2 (–3·7 to 3·2)	0·893
Body composition						
	Fat mass (g)		2056 (714), n=134	2128 (762), n=135	–72 (–250 to 105)	0·423
	Lean + BMC mass (g)		6182 (805), n=134	6265 (922), n=135	–84 (–292 to 124)	0·428
	Total mass (g)		8234 (1129), n=134	8427 (1208), n=135	–193 (–474 to 88)	0·178
	Percent fat (%)		24·5 (6·8), n=134	25·3 (6·7), n=135	–0·8 (–2·4 to 0·8)	0·329
BMC(g)			186·0 (35·7), n=135	191·8 (32·9), n=135	–5·7 (–13·9 to 2·5)	0·173
Bone mineral density (g/cm^2^)			0·25 (0·03), n=135	0·25 (0·03), n=135	–0·001 (–0·008 to 0·006)	0·770
Lipid profile (mg/dL)						
	Total cholesterol		141·4 (33·1), n=161	141·8 (32·9), n=173	–0·4 (–7·5 to 6·7)	0·919
	Triglycerides		123·4 (61·6), n=160	125·1 (67·8), n=173	–1·6 (–15·6 to 12·4)	0·818
	HDL		37·9 (12·3), n=161	38·3 (12·1) n=173	–0·4 (–3·1 to 2·2)	0·758
	LDL		77·3 (27·9), n=160	76·8 (28·0), n=172	0·5 (–5·5 to 6.5)	0·870
	VLDL		25·8 (12·1), n=160	26·3 (13·7), n=173	–0·5 (−3·3 to 2·3)	0·731
HOMA-IR*			0·4 (0·3–0·7), n=153	0·4 (0·2–0·7), n=166	..	0·675
Blood pressure (mm Hg)						
	Systolic		80·9 (6·6), n=135	80·9 (6·2), n=149	0·05 (–1·4 to 1·5)	0·951
	Diastolic		50·1 (5·8), n=135	50·4 (5·3), n=149	–0·3 (–1·6 to 1·0)	0·632
Serum ferritin (μg/dL)			5·4 (3·2–12·4), n=160	5·7 (2·5–13·3), n=173	..	0·732
Serum ferritin <12 μg/dL, n (%)			119/160 (74·4%)	126/173 (72·8%)	1·0 (0·9 to 1·2)	0·750

Data are mean (SD), n/N (%), or median (IQR). p values are from Fisher-exact test, t-test, Wilcoxon rank-sum or **χ^2^** test. IR=incidence rate. LRTI=lower respiratory tract infection. MoDQ_50_=motor developmental quotient–50th centile. MoDQ_97_=motor developmental quotient–97th centile. MeDQ_50_ =mental developmental quotient–50th centile. BMC=bone mineral content. VLDL=very low density lipoprotein. HOMA-IR=Homeostatic Model Assessment for Insulin Resistance.

* Outcome of death was known for all enrolled infants except for one infant in the 4 month group.

† Outcome of hospital admission was known for 188 infants in 4 month group (184 who completed follow-up and 4 who died), and for 192 infants in the 6 month group (189 who completed follow-up, 2 who died, and 1 infant who was lost to follow-up but had a documented episode of hospital admission before being lost).

‡ This is incidence rate ratio. Primary outcome and all secondary outcomes except deaths and hospital admissions were measured at 12 months corrected age. Death and hospital admission data is for the entire study duration, ie, from enrolment at 4 months corrected age until final outcome assessment at 12 months corrected age.

**Table 3:** Prespecified subgroup analysis for primary outcome, weight-for-age *Z* score at 12 months of corrected age

	**4 month group**	**6 month group**	**Mean difference (95% CI)**	**p value**
**Study site**				
All India Institute of Medical Sciences	–1·5 (1·3), n=57	–1·21 (1·4), n=54	–0·3 (–0·8 to 0·2)	0·306
Safdarjung Hospital	–1·7 (1·1), n=119	–1·75 (1·2), n=122	0·05 (–0·2 to 0·3)	0·743
Kasturba Hospital	–1·4 (1·1), n=9	–2·1 (1·0), n=13	0·7 (–0·2 to 1·6)	0·110
**Intrauterine fetal growth category**				
Small for gestational age	–2·1 (1·2), n=52	–2·3 (1·2), n=50	–0·2 (–0·3 to 0·7)	0·408
Appropriate for gestational age	–1·4 (1·1), n=131	–1·4 (1·2), n=137	–0·03 (–0·3 to 0·2)	0·806
**Gestational age at birth**				
<28 weeks	–2·0 (1·1), n=4	–2·2 (1·9), n=6	0·2 (–2·2 to 2·7)	0·836
28–30 weeks	–1·7 (1·2), n=58	–1·7 (1·4), n=67	–0·04 (–0·5 to 0·4)	0·856
31–33 weeks	–1·5 (1·1), n=122	–1·5±1·1, n=116	0·002 (–0·3 to 0·3)	0·987
**Feeding at complementary feeding randomisation**				
Breastfed	–1·7 (1·1), n=95	–1·8 (1·2), n=93	0·1 (–0·2 to 0·4)	0·561
Non-breastfed	–1·7 (1·4), n=33	–1·6 (1·4), n=42	–0·06 (–0·7 to 0·6)	0·846
Mixed food	–1·5 (1·1), n=55	–1·4 (1·3), n=54	–0·1 (–0·5 to 0·4)	0·672

Data are mean (SD) unless otherwise specified. p values are from Student *t* test

**Table 4:** Dietary indicators at 12 month corrected age

		**4 month group**	**6 month group**	**P-value**
Bottle feeding		100/183 (54·6%)	110/189 (58·2%)	0·489
Number of food groups offered		3·7 (0·9), n=183	3·5 (1·0), n=189	0·037
Acceptable minimum dietary diversity*		110/183 (60·1%)	104/189 (55·0%)	0·321
Meal frequency				
	Breastfed infants (includes only non-liquid feeds)	4·2 (1·4), n=123	4·3 (1·6), n=118	0·762
	Non-breastfed infants (includes both milk feeds and solid or semisolid feeds)	8·5 (2·1), n=60	9·0 (2·0), n=71	0·138
Acceptable minimum meal frequency				
	Breastfed infants (≥3)	112/123 (91·1%)	104/118 (88·1%)	0·457
	Non-breastfed infants (≥4)	59/60 (98·3%)	71/71 (100%)	0·279
Minimum acceptable diet (%)†		108/184 (58·7%)	103/189 (54·5%)	0·406
Consumption of iron-rich or iron-fortified foods (%)		175/184 (95·1%)	174/189 (92·1%)	0·216
Consistency of complementary food-thick		182/182* (100%)	182/187* (97·3%)	0·085
Texture of complementary food-grainy		166/182 (91·2%)	167/187 (89·3%)	0·538

Data presented as number (%) or mean (SD) unless otherwise specified. P value is from χ^2^ test or Student t test.

* Proportion of infants receiving food from four or more food groups.

† Breastfed infants who received food from four food groups or more, and solid, semisolid, or soft foods at least three times on previous day; non-breastfed infants who received at least 2 milk feeds, food from four food groups or more (not including milk), and 4 or more meals during the previous day.
