# Correction to *Lancet Glob Health* 2017; 5: e501–11

**DOI:** 10.1016/S2214-109X(18)30188-8

**Published:** 2018-04-10

*Gupta S, Agarwal R, Aggarwal KC, et al. Complementary feeding at 4 versus 6 months of age for preterm infants born at less than 34 weeks of gestation: a randomised, open-label, multicentre trial.* Lancet Glob Health *2017; **5:** e501–11—*In this Article, the last sentence of the Procedures section should have read “Z scores for weight, length, head circumference, weight for length, and body–mass index at subsequent timepoints, (ie, 4, 5, 6, 7, 9, and 12 months corrected age) were calculated using WHO-MGRS growth standards.” In Figure 1, the box describing patients who did not receive allocated intervention in the 6 month group should have included “1 did not start complementary feeding until 12 months” and the box describing patients who did not receive allocated intervention in the 4 month group should have included “1 did not start complementary feeding before death, which occurred between 5 and 6 months”. These corrections have been made as of April 10, 2018.

