# Supplementary material for: Complementary feeding at 4 versus 6 months of age for preterm infants born at less than 34 weeks of gestation: a randomised, open-label, multicentre trial
Source: Lancet Glob Health. 2017 Apr 7;5(5):e501–11. doi: 10.1016/S2214-109X(17)30074-8 (PMC5388893; doi:10.1016/S2214-109X(17)30074-8)
Supplement: Supplementary appendix [file mmc1.pdf]

THE LANCET
Global Health

Supplementary appendix

This appendix formed part of the original submission and has been peer reviewed. We post it as supplied by the authors.

Supplement to: Gupta S, Agarwal R, Aggarwal KC, et al. Complementary feeding at 4 versus 6 months of age for preterm infants born at less than 34 weeks of gestation: a randomised, open label, multi-centre trial. *Lancet Glob Health* 2017; **5:** e501–11.

Web Appendix

Panel 1. Rationale for choosing 4 vs. 6 months of corrected age

A recent ESPGHAN review suggests that in term infants, CF should not be started before 17 weeks (~4 months) of age, and should not be delayed beyond 26 weeks (~6 months) of age.[1] Besides the age at initiation being 4 or 6 months, for preterm infants, we also need to answer whether this age should be taken as "corrected" age (CA; age adjusted for period of prematurity), or "postnatal" age (PNA; age since birth). Since CA is most often used to assess growth (anatomical development) and neurodevelopment (functional development) of preterm infants until two-three years of age, it seems logical that their nutritional needs, which parallel growth, and the anatomical and functional maturity of the gastrointestinal tract should also be guided by CA.

Reference:

1. Agostoni C, Decsi T, Fewtrell M, et al. ESPGHAN Committee on Nutrition. Complementary feeding: a commentary by the ESPGHAN Committee on Nutrition**.** J Pediatr Gastroenterol Nutr 2008 Jan;46(1):99-110

	Site 1: AIIMS	Site 2: SJH	Site 3: KH
**Type of institute**	Autonomous healthcare and research institute	Urban, central government run institute	District level first referral health facility
**Deliveries per year**	2500	25000	12000
**Patients' primary socio-economic strata**	Middle to upper middle	Lower middle to lower	Lower to very low
**Neonatal intensive care unit**			
Level of care	Tertiary inborn	Tertiary inborn	Tertiary Inborn
Number of beds	18	50	30
Nurse: patient ratio	1:2 to 1:3	1:6	1:10
Average bed occupancy	90%	120%	110%
Overall survival rate (2014)	86·1%	85·2%	93.2%
**Antenatal details of delivering mothers**			
Number of antenatal visits	Most receive ≥3 visits	About 70% receive ≥3 visits	About 50% receive ≥3 visits
Iron-folic acid and calcium-vitamin D supplementation received	More than 80%	More than 80%	More than 80%
Management for any complicating conditions	Appropriate	Appropriate	Appropriate
Reliable information on date of last menstrual period (LMP) and first trimester ultrasound	Available in almost 100%	Available in approx 70%	Available in approx 60%
Antenatal steroid coverage	High	High	High
**Clinical practices with respect to preterm infants <34 wks of gestation**			
Intensive care	Routinely cared for in the intensive care unit	Routinely cared for in the intensive care unit	Routinely cared for in the intensive care unit
Total parenteral nutrition	Practiced	Not practiced	Not practiced
Minimal enteral nutrition	Practiced	Practiced	Practiced
Expressed breast milk (EBM) feeding by gavage/paladai	Practiced	Practiced	Practiced
Exclusive breastfeeding	Actively pursued and promoted as part of unit policy However, formula milk is provided based on clinical need		
Human milk banking	Not practiced	Not practiced	Not practiced
Post discharge care	Provided through a dedicated weekly high risk follow up clinic		
Feeding after discharge	Exclusive breastfeeding followed by EBM by katori-spoon as required. Formula or other milk supplementation only in cases of lactation failure or inappropriate weight gain.		
Micronutrient supplementation policy	Vitamin D 400 IU/day (800 IU /day for infants <1500g) Iron at 2-3 mg/kg per day beginning at 2-4 weeks of postnatal age till one year of correctedage.	Vitamin D 400 IU/day ; Calcium 164 mg/day (approx 80-100mg/kg/day) Iron at 2-3 mg/kg per day beginning at 2-4 weeks of postnatal age till one year of corrected age.	
Others- ROP screening, cranial USG	Routine, serial CUS during nursery stay followed by one at 40 wks if required		
Post-discharge early intervention services	Hospital based, routinely provided		Not available

Table 1. **Site description**

Procedures	Personnel involved	Methods
**Tracking from birth till discharge**		
Daily tracking of NICU admissions	Research team	Through daily record of NICU admission register
Gestation assessment	Residents, verified by SG (AIIMS); SG (SJH and KH)	LMP/I trimester ultrasound and corroboration with ENBS done within 96 hours of birth
Morbidity recording during birth hospitalization	Research team	Through daily record of NICU admission register
Parental/family contact<br>Discharge advice	Research team<br>Residents at all sites; research team met to record contact details	Through daily visit to mother, and need based counseling<br>Advice on post discharge care (temperature, hygiene, feeding, danger signs and follow up)
**Post discharge tracking and follow up**		
Clinical follow up schedule	Residents/parent/family driven	**AIIMS:** 2-7 days after discharge, then every 2 weeks (<32wks/<1500g), then monthly. Infants ≥32 wks or ≥1500g followed up once at 6 wks of age and only those with any high risk factors were followed monthly thereafter. Individulaized as per infants needs at any stage.<br>**SJH and KH:** 2 weeks after discharge then individualized as per infants' needs
Post discharge telephonic contact	Research team	**AIIMS:** Telephonic contact 2 weeks prior to 4 month corrected age. Home visit if required<br>**SJH/KH:** Telephonic contact made 6 weeks after discharge to call for research visit by dedicated research team to ensure follow up. This was later changed to 2 weeks after one year of study
Post discharge follow up care	Research team	Same as described earlier
Feeding and micronutrient supplementation	Residents (AIIMS)<br>Research team at SJH and KH	Same as described earlier
**Enrolment and randomization (at 4 month corrected age)**		
Informed consent and randomization	SG	Witten informed consent allocation concealment and randomization process described in text
Counseling for initiation of CF or continue milk feeding till 6 months of age, micronutrient supplementation	SG	Using standardized counseling package described in text
Oro-motor and developmental assessment	SG, assisted by early intervention professionals	Early intervention initiated in all infants if required in both groups, as per standard of care
Anthropometry	SG	Using standard procedures
Baseline DXA	Technicians from Department of Radio-diagnosis at AIIMS	Using standard procedures
Baseline data recording	SG	-
**Post enrolment follow up**		
Telephonic calls on day 2, 7, 14 and day 21 in both groups	Medical social worker	Inquiry with respect to adherence to recommended feeding advice, referral to PI for defaulters, morbidity inquiry
5 month visit	Medical social worker and dietician	Home/hospital visit: 24 hour dietary recall, anthropometry, dietary advice
6 month visit	SG: Counseling for initiation of complementary food to 6 month group<br><br>Medical social worker and dietician	Hospital visit for 6 month group for counseling on initiation of complementary feeding (done telephonically in an occasional case if hospital visit was not possible)<br><br>24-hr dietary recall, anthropometry, dietary advice to all through home/hospital visit
Telephonic calls on day 2, 7, 14 and day 21 in both groups	Research team	Inquiry with respect to adherence to recommended feeding advice, referral to PI for defaulters, morbidity inquiry
7 month visit	Medical social worker and dietician	Home/hospital visit: 24 hour dietary recall, anthropometry, dietary advice
9 month visit	Medical social worker and dietician	Home/hospital visit: 24-hr dietary recall, anthropometry, dietary advice
Telephonic inquiry at 10, 11 months	Medical social worker	Inquiry for any incident morbidity
12 month visit	Nurse, clinical psychologist, physiotherapist (all masked to group assignment)	Anthropometry, blood sampling, DXA, neurodevelopment

NICU-Neonatal intensive care unit, SG-First author, AIIMS-All India Institute of Medical Sciences, SJH-Safdarjung hospital, KH-Kasturba hospital, LMP-Date of last menstrual period, ENBS-Expanded New Ballard Scoring, DXA-Dual Energy X-Ray Absorptiometry

***Table 2.* Summary of study procedures**

Panel 2. Method of doing 24-hour dietary recall

The 24-hour dietary intake assessments were performed by a trained and experienced dietician. The method of obtaining the 24-hour has been tested in the paediatric and neonatal clinical and ward settings by senior dieticians in the paediatric department of our tertiary academic healthcare and research centre.

The dietician conducted a structured interview with the mother (or caregiver) asking her to recall all food and drinks provided to the child during the previous 24 hours, beginning with the first feed in the morning. She asked the mother to recall chronologically the time and associated events around feeding the child for better recall. Specific probes were used with respect to the commonly used infant foods which might have been forgotten, or any foods that infant might have received as part of the common family meals.

Details were obtained with respect to the ingredients used to prepare a particular food-the dietician weighed the amount of raw ingredients in case the mother was only able to indicate the amount used using her fist or any other home utensil. The raw ingredients used to prepare a recipe were then converted to standard cooked weights/portion sizes using standard Indian references. The dietician cross-verified the conversion of raw ingredients to the cooked weights/portion sizes by asking the mother the total cooked quantity prepared, and measured the utensils she used to ascertain the cooked weight. She then asked the mother the amount eaten by the child and also measured the utensils used for feeding the child as required, to cross-verify the amount consumed by the child. The nutritional value of the food consumed by the child were derived using the nutritive values of Indian foods. All details were noted on a structured 24-hour dietary recall record form, and verified by an independent expert.

Panel 3. Definition of dietary indicators

Acceptable minimum dietary diversity: The proportion of infants at 12 months corrected age who received foods from four or more of the seven food groups during the previous day on the 24-hour dietary recall. The seven food groups were: grains, roots and tubers, legumes and nuts, dairy products (milk, yogurt, cheese), flesh foods (meat, fish, poultry and liver/organ meats), eggs, vitamin-A rich fruits and vegetables, and other fruits and vegetables.

Acceptable minimum meal frequency: The proportion of breastfed infants who received solid, semi-solid, or soft foods at least 3 times or more; or, if non-breastfed, received milk solid, semi-solid, or soft foods at least four times or more in the previous 24-hours on the 24-hour dietary recall.

Minimum acceptable diet: This is a composite indicator based on minimum dietary diversity and minimum meal frequency, calculated differently for breastfed and non-breastfed infants.

- Breast-fed infants are defined to have received minimum acceptable diet if they have been provided semisolid/soft/solid foods at least 3 times in the previous day from four or more of the seven food groups listed above in previous 24-hours.
- Non-breastfed infants are defined to have received minimum acceptable diet if they have received at least 2 milk feedings, food from four or more food groups (excluding milk feeds), and have been fed at least four times in previous 24-hours.

Consumption of iron-rich or iron-fortified foods: This was defined as proportion of children who received an iron-rich food or iron-fortified food (e.g. commercially available cereals) or a food that was fortified in the home with a product that included iron during the previous day.

Results: Additional data

CA (month)	Any breastfeeding			Any complementary feeding		
	4-month	6-month	P	4-month	6-month	P
4	165/203 (81·3%)	153/197 (77·7%)	0·370	0	0	NA
5	158/197 (80·2%)	134/180 (74·4%)	0·181	188/197 (95·4%)	0/180	<0·001
6	145/191 (75·9%)	137/186 (73·7%)	0·613	175/191 (91·6%)	6/186 (3·2%)	<0·001
7	134/183 (73·2%)	127/183 (69·4%)	0·419	179/183 (97·8%)	166/183 (90·7%)	<0·003
9	126/181 (69·6%)	121/175 (69·1%)	0·923	177/181 (97·8%)	169/175 (96·6%)	0·487
12	123/183 (67·2%)	118/189 (62·4%)	0·335	182/183 (98·9%)	187/189 (98·9%)	0·978

Data are n (%). P-value from chi-square test. CA-Corrected age

Table 3. **Dietary profile of study infants over time**

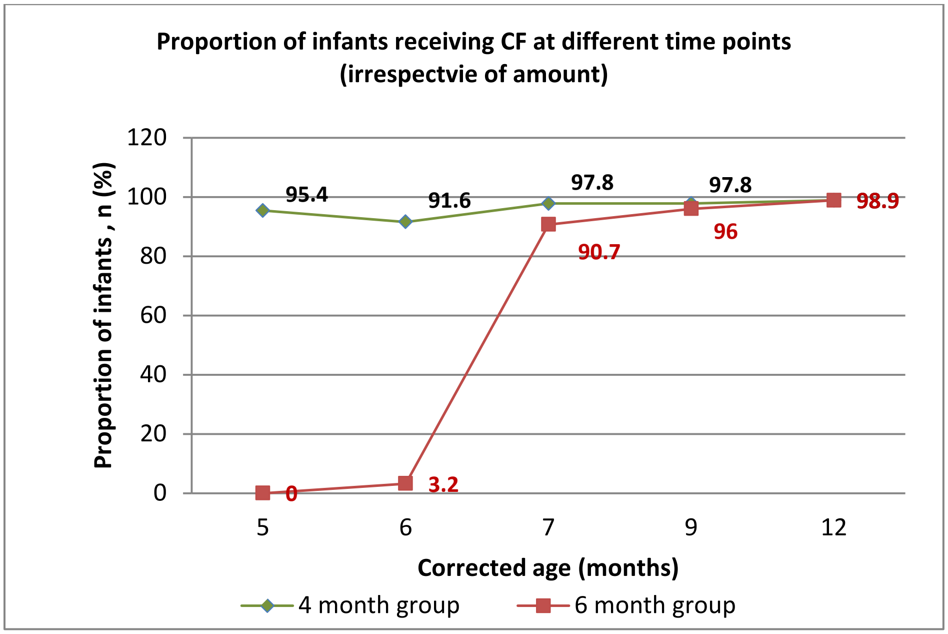

Figure 1. **Proportion of infants receiving complementary foods (irrespective of amount) at different time points by study group**

Growth. Mean weight (7794 vs. 7846 g, p=0·65) and weight for age z-scores were comparable at 12 months CA as well as at all the intermediary time points (table 4). Mean values of length (71·1 vs. 71·1 cm), head circumference (43·7 vs. 43·8 cm), body mass index (10·9 vs. 11·0 kg/m^2) and their respective z-scores were comparable between the two groups at 12 months CA (tables 4-6). Additionally, the change in weight, length, head circumference and body mass index and their respective z scores from birth until 12 months CA was also comparable between the two groups. Only a small proportion of infants exhibited catch up growth (increase of >0·67 SDS) in weight for age (10·8 vs. 5·8%), length for age (18·0 vs. 11·9%) and head circumference for age (10·2 vs. 4·7%) from birth until 12 months CA (table 7).

CA (months)	n		Weight (g)		P	Weight for age z-score		P
	4-month	6-month	4-month	6-month		4-month	6-month	
4 (randomization)	204	197	5117±906	5187±928	0·446	-2·3±1·4	-2·2±1·4	0·711
5	197	180	5681±968	5652±958	0·771	-2·0±1·4	-2·1±1·4	0·658
6	191	186	6144±1018	6152±972	0·942	-1·9±1·4	-1·9±1·4	0·908
7	183	183	6483±1008	6576±983	0·375	-1·8±1·3	-1·8±1·3	0·707
9	181	175	7071±1016	7138±1090	0·555	-1·7±1·2	-1·7±1·3	0·914
12	184	189	7794±1067	7846±1144	0·648	-1·6±1·2	-1·6±1·3	0·965

Data are mean±SD; p-value from Student t-test. CA-Corrected age

***Table 4.* Weight and weight for age z-scores among study infants at different time points by study group**

CA (months)	n		Length (cm)		P	Length for age z score		P
	4-month	6-month	4-month	6-month		4-month	6-month	
4 (randomization)	204	195	58·5±3·1	58·6±3·2	0·778	-2·0±1·4	-2·0±1·5	0·918
5	196	179	60·7±3·2	60·6±3·3	0·751	-1·8±1·4	-1·9±1·5	0·634
6	188	183	63·0±3·3	62·9±3·1	0·751	-1·7±1·4	-1·8±1·4	0·562
7	177	180	64·8±3·3	64·9±3·1	0·947	-1·4±1·4	-1·5±1·3	0·522
9	178	174	67·5±3·2	67·5±3·2	0·986	-1·5±1·3	-1·6±1·4	0·695
12	184	189	71·1±3·0	71·1±3·3	0·813	-1·6±1·2	-1·6±1·3	0·491

Data are mean±SD. P-value from Student t-test. CA-corrected age

***Table 5*. Length and length for age z scores among study infants at different time points by study group**

CA (months)	n		Head circumference (cm)		P	Head circumference for age z-score		P
	4-month	6-month	4-month	6-month		4-month	6-month	
4 (randomization)	204	197	38· 9±1·6	39·0±1·4	0·315	-1·7±1·2	-1·7±1·2	0·647
5	196	180	39·9±1·4	39·9±1·3	0·944	-1·6±1·1	-1·7±1·1	0·841
6	188	183	40·8±1·4	40·8±1·3	0·878	-1·5±1·1	-1·6±1·0	0·787
7	177	180	41·5±1·4	41·6±1·3	0·633	-1·4±1·1	-1·5±1·0	0·665
9	178	174	42·5±1·4	42·6±1·3	0·436	-1·5±1·0	-1·5±1·0	0·858
12	184	189	43·7±1·5	43·8±1·3	0·477	-1·4±1·1	-1·3±1·0	0·941

Data are mean±SD. P-value from Student t-test. CA-corrected age

***Table* 6. Head circumference and head circumference for age z scores among study infants at different time points by study group**

CA (months)	n		BMI (kg/m^2)		P	BMI for age z score		P
	4-month	6-month	4-month	6-month		4-month	6-month	
4 (randomization)	204	195	8·7±1·2	8·8±1·2	0·471	-1·5±1·2	-1·5±1·2	0·571
5	196	179	9·3±1·2	9·3±1·2	0·677	-1·3±1·2	-1·4±1·2	0·634
6	188	183	9·7±1·3	9·7±1·2	0·824	-1·3±1·3	-1·3±1·2	0·803
7	177	180	10·0±1·2	10·1±1·2	0·242	-1·3±1·2	-1·2±1·2	0·279
9	178	174	10·4±1·1	10·5±1·3	0·393	-1·2±1·1	-1·1±1·2	0·535
12	184	189	10·9±1·2	11·0±1·2	0·488	-1·0±1·0	-0·9±1·1	0·561

Data are mean±SD. P-value from Student t-test. CA-corrected age

***Table* 7. Body mass index (BMI) and BMI for age z scores among study infants at different time points by study group**

	4-month	6-month	P
**Birth to 12 month CA***			
Weight	20/184 (10·9%)	11/189 (5·8%)	0·077
Length	10/50 (20·0%)	5/42 (11·9%)	0·295
Head circumference	5/49 (10·2%)	2/43 (4·7%)	0·316
**40wks PMA to 12 month CA***			
Weight	101/163 (62·0%)	95/168 (56·5%)	0·372
Length	45/95 (47·4%)	43/92 (46·7%)	0·931
Head circumference	45/161 (28.0%)	36/166 (21·7%)	0·190
**4 -12 month CA***			

Weight	80/184 (43.5%)	72/189 (38·1%)	0·290
Length	64/184 (34·8%)	61/187 (32·6%)	0·660
Head circumference	56/184 (30·4%)	51/189 (27·0%)	0·461
Weight for length	39/184 (21·2%)	37/186 (19·9%)	0·75

*Denominator includes infants whose data were available at both time points. Data are n (%). P-values from chi-square test. CA-Corrected age, PMA-Postmenstrual age

***Table 8.* Proportion of infants showing catch up growth (ΔSDS of 0·67) in the respective parameters during specified periods**

Dietary data. Dietary calorie intake per kg body weight (excluding intake from breast milk) was significantly higher in the 4-month group until seven months CA, and comparable thereafter to that in the 6-month group, while protein intake was higher until nine months CA and comparable thereafter (table 4). Only two-third of the dietary energy intake in either group came from CF at 12 months CA, with rest being from milk (other than breast-milk) (table 4, appendix table 9)

High proportion of infants were receiving iron (4-month CA: 91·4% vs. 88·9%, p=0·42; 12 months CA: 71·6% vs. 70·9%, p=0·88), and vitamin D (4-month CA: 88·9% vs. 90·0%, p=0·71; 12 months CA: 69·4% vs. 70·9%, p=0·75) (appendix table 10). Mean total iron intake from supplements and food was 2·4 vs. 2·3 mg/kg/day in 4- and 6-month groups respectively at five months CA, which decreased to 1·8 and 1·9 mg/kg/day, respectively by 12 months CA. Proportion of iron intake from CF remained low with a mean of 0·2 mg/kg/day at 12 months CA (table 4).

			ENERGY INTAKE FROM CF: PROPORTION OF TOTAL ENERGY INTAKE			PROTEIN INTAKE FROM CF: PROPORTION OF TOTAL PROTEIN INTAKE		
CA (mo)	**4-month** N	**6-month** N'	**4-month** Median (IQR)	**6-month** Median (IQR)	**P**	**4-month** Median (IQR)	**6-month** Median (IQR)	**P**
5	197	180	62 (30-100)	0 (0-0)	<0·001	47·1 (18·8-100)	0	<0·001
6	191	186	64 (34-100)	0 (0-0)	<0·001	53·3 (22·7-100)	0	<0·001
7	183	183	67 (43-100)	43 (20-100)	<0·001	56·3 (30·9-100)	30·6 (11·1-100)	<0·001
9	181	175	65 (44-100)	59 (33-100)	0·140	54·2 (31·9-100)	45·5 (23·4-100)	0·051
12	183	189	66 (44-100)	63 (42-100)	0·558	59·4 (34·6-99·8)	53·7 (33·3-100)	0·692

***Table 9.* Energy and protein intake from complementary food as proportion of total dietary energy and protein intakes respectively**

CA (months)	Iron			Calcium			Vitamin D		
	4-month	6-month	P	4-month	6-month	P	4-month	6-month	P
5	180/197 (91·4%)	160/180 (88·9%)	0·42	116/197 (58·9%)	109/180 (60·6%)	0·741	175/197 (88·9%)	162/180 (90·0%)	0·713
6	171/191 (89·5%)	169/186 (90·9%)	0·79	103/191 (53·9%)	106/186 (57·0%)	0·550	169/191 (88·5%)	172/186 (92·5%)	0·187
7	163/183 (89·1%)	166/183 (90·7%)	0·60	109/183 (59·6%)	104/183 (56·8%)	0·596	167/183 (91·3%)	167/183 (91·3%)	1·00
9	153/181 (84·5%)	146/175 (83·4%)	0·78	94/181 (51·9%)	85/175 (48·6%)	0·526	148/181 (82·3%)	140/175 (80·0%)	0·671
12	131/184 (71·2%)	134/189 (70·9%)	0·95	84/184 (45·7%)	81/189 (42·9%)	0·59	127/184 (69·0%)	134/189 (70·9%)	0·752

Data are number (%). CA-Corrected age

***Table 10.* Proportion of study infants receiving supplements at different points of time**

	All infants					Breastfed infants					Non breastfed infants				
CA (mo)	4-month		6-month		P	4-month		6-month		P	4-month		6-month		P
	N	Median (IQR)	N	Median (IQR)		N	Median (IQR)	N	Median (IQR)		N	Median (IQR)	N	Median (IQR)	
5	197	67 (35-120)	180	27 (0-93)	<0·001	158	53 (28-99)	134	0 (0-40)	<0·001	39	147 (109-194)	46	117 (93-145)	0·072
6	191	73 (40-121)	186	30 (0-98)	<0·001	145	57 (32-90)	137	0 (0-40)	<0·001	46	137 (119-170)	49	108 (81-129)	<0·001
7	183	82 (52-118)	183	65 (34-105)	<0·001	134	67 (46-95)	127	44 (25-71)	<0·001	49	118 (96-150)	56	108 (91-147)	0·381
9	181	84 (55-117)	175	80 (41-114)	0·108	126	69 (48-92)	121	55 (36-85)	<0·008	55	117 (96-149)	54	119 (101-143)	0·964
12	183	86 (68-107)	189	82 (60-112)	0·244	123	79 (59-96)	118	66 (50-85)	0·030	60	108 (87-130)	71	108 (87-132)	0·884

Table 11*. Dietary Energy intake per kg in study infants at different time points by study groups (Excludes energy intake from breast milk)*
